# Recombinant TSH and Lithium Overcomes Amiodarone-Induced Low Radioiodine Uptake in a Thyrotoxic Female

**DOI:** 10.5812/ijem.5406

**Published:** 2012-09-30

**Authors:** Nestor Eric R. Laplano, Leilani B. Mercado-Asis

**Affiliations:** 1 Section of Endocrinology and Metabolism, University of Santo Tomas Hospital, Manila, Philippines

**Keywords:** rhTSH, Lithium, Amiodarone

## Abstract

**Introduction:**

Recombinant human thyroid-stimulating hormone(rhTSH) increases radioactive iodine uptake(RAIU) in selected populations, while lithium is used as an adjunct to radioactive iodine (RAI) therapy in Graves’ disease with low RAIU. In this report, both drugs used in combination, overcame low iodine-131 uptake in a Graves’ patient.

**Clinical Case:**

A 39-year old female with Graves’ disease, acquired thionamide-induced agranulocytosis, and severe hypokalemia, subsequently went into cardiorespiratory arrest. On resuscitation, she had ventricular tachyarrhythmias which were cardioverted using amiodarone. She was subsequently placed on IV hydrocortisone amiodarone and propranolol. On admission, she was normotensive, tachycardic, and afebrile. She had fine tremors, hyper reflexia, and diffuse, non-tender thyromegaly. Initial investigations showed normal complete blood count, hypokalemia and elevated alanine transaminase levels. Levels of thyroid stimulating hormone were low (0.03 uIU/L, N = 0.27-3.75). Thyroid ultrasound showed diffuse thyromegaly with uniform echopattern and normal color flow Doppler, radioiodine uptake showed low uptake at 0400h and 2400h (6% and 7%, respectively). In preparation for RAI therapy, she was given lithium 900mg/day for 12 days to increase RAI retention. To increase iodine-131 uptake, two doses of 0.9mg rhTSH were injected intramuscular, 24 hours apart, before RAI therapy. Repeat RAIU after the second dose of rhTSH showed more than a 5-fold increase in 0400h uptake compared with the baseline (32% vs. 6%). Exactly 24 hours after the second dose of rhTSH, she was given 25mCi of iodine-131. Thereafter, the patient’s clinical and biochemical markers continued to improve. She became hypothyroid and is currently on levothyroxine replacement therapy.

**Conclusions:**

This case demonstrates the efficacy of combining rhTSH and lithium to overcome amiodarone-induced low iodine-131 uptake in Graves’ disease.

## 1. Introduction

Radioiodine therapy is well-recognized as an effective treatment for hyperthyroidism. The goal of therapy is to destroy sufficient thyroid tissue to render the patient either euthyroid or hypothyroid (1). Fixed dose regimens and dosimetric calculations using thyroid volume and radioactive iodine uptake (RAIU) are used to determine the effective dose to cure hyperthyroidism. However, for a subset of thyrotoxic patients who are on amiodarone therapy for dangerous cardiac arrhythmias, and who are poor surgical candidates, such iodine-rich medications may interfere with RAIU, and iodine-131 treatment may be unsuccessful.

Recently, lithium has been considered to be a useful adjunct to radioactive iodine (RAI) therapy in patients with Graves’ disease who demonstrate low RAIU (2). Lithium increases RAI retention by reducing its release from the thyroid gland, thus promoting faster control of thyrotoxicosis and avoidance of transient exacerbation (due to methimazole withdrawal and RAI administration), without a concomitant effect on thyroidal RAIU (3-5).

Recombinant human thyroid-stimulating hormone (rhTSH) promotesiodine-131 uptake and thyroglobulin production in thyroid remnants and metastatic thyroid cancer patients remaining on thyroid hormone replacement. This provides a beneficial alternative to thyroid hormone withdrawal, in the evaluation of thyroid cancer persistence and its recurrence (6). Administration of rhTSH also; increases iodine-131uptake in nontoxic nodular goiter (7, 8), induces a more homogenous distribution of RAI by stimulating uptake in relatively cold areas (9), and allows a reduction in radiation doses with retained efficacy (10, 11). The utility of rhTSH in increasing RAIU in iodine-loaded individuals (iodide-treatment and amiodarone-induced thyrotoxicosis (AIT) type 1, has been reported previously, albeit with caution (12). Here, we demonstrate the use of rhTSH in combination with lithium to overcome low iodine-131 uptake in a thyrotoxic patient given amiodarone for potentially fatal arrhythmias.

## 2. Patients and Methods

The patient was a39year old female who presented witha one year history of heat intolerance, weight loss, palpitations and easy fatigability. She was diagnosed with Graves’ disease and was prescribed with methimazole (40 mg/day) and propranolol (60 mg/day). After three weeks of therapy, she developed a fever, sore throat and pancytopenia. She was diagnosed with thionamide-induced agranulocytosis, accompanied by severe hypokalemia, hypomagnesemia, and hypocalcemia. TSH was suppressed at < 0.05 uIU/mL, while free T4 (63.89 pmol/L) and free T3 (11.5 pmol/L) levels were elevated. She was started on hydrocortisone (400 mg/day) and propranolol (120 mg/day), and electrolyte abnormalities were addressed. However, she developed ventricular tachyarrhythmias and subsequently went into cardiorespiratory arrest. She was eventually resuscitated after 30minutes and successful cardio version was achieved using amiodarone. She was maintained on IV hydrocortisone (150 mg/day), propranolol (60 mg/day PO),amiodarone (200 mg/day PO), and oral potassium replacement. Once hemo dynamically stable, she was airlifted for transfer to our institution for definitive management.

On examination, she was hyposthenic (BMI 18 kg/m ^2^), normotensive (BP 110/70 mmHg), afebrile (37.1oC), tachypneic (RR 24 breaths/min) and tachycardic (HR 112 beats/min, regular rhythm). She had warm, moist skin, fine finger tremors, hyperreflexia, anicteric sclera, and diffuse, smooth, non-tender, firm thyromegaly measuring 9cm x 5cm, with no bruit. Initial investigations showed normal complete blood count, hypokalemia (3.51mmol/L, n = 3.7-5.0), hypocalcemia (1.13mg/dL, n = 1.18-1.32), and slightly elevated alanine aminotransferase (ALT) (40.76 U/L, n = up to 31). An electrocardiogram showed normal sinus rhythm with diffuse T-wave inversion and cardiac enzymes were negative. Two-dimensional echocardiogram showed left ventricular hypertrophy with good wall motion and contractility and normal ejection fraction. A radioimmunoassay (Turbo TSH, Hungary) showed that her TSH was suppressed (0.03 uIU/mL, n = 0.27-3.75) with normal free T4 (13.5 pmol/L, NV 10.3-25.7) and low free T3 (1.8 pmol/L, NV 2.2-6.8). Thyroid ultrasound (Aloka Co. Japan) with color flow Doppler (CFD) showed diffuse thyromegaly (right lobe: 5.2 x 1.3 x 1.7cm; left lobe: 5.2 x 1.4 x 1.8cm) with uniform echo pattern and normal CFD.

After oral administration of a tracer activity of 2MBq iodine-131, RAIUwas measured as a percentage of the administered dose, corrected for physical decay, at 0400h and 2400h using a collimated scintillation probe (Atom-Lab Uptake Machine, Biodex Medical Systems, Shirley, NY).Both RAIU measurements were low at 6% at 0400h (NV 13-22)and 7% at 2400h (NV 25-80).

Five days prior tothe contemplated RAI therapy; she had been started on lithium 900 mg/day. Two days before RAI therapy, freeze dried rhTSH (Thyrogen, Genzyme Transgenic Corp., Cambridge, MA) in ampoules containing 0.9 mg rhTSH was reconstituted in 1.2 mL sterile water and injected to both gluteus muscles 24 hours apart. Lithium was continued to complete the 12 days of therapy, and serum lithium measurements were maintained at normal levels.

Repeat RAIU after the second dose of rhTSH showed more than a 5-fold increase in uptake (32% vs. 6%) at 0400h. Exactly 24 hours after the second dose of rhTSH, she was given 25MCi of iodine-131.

**Figure 1:**
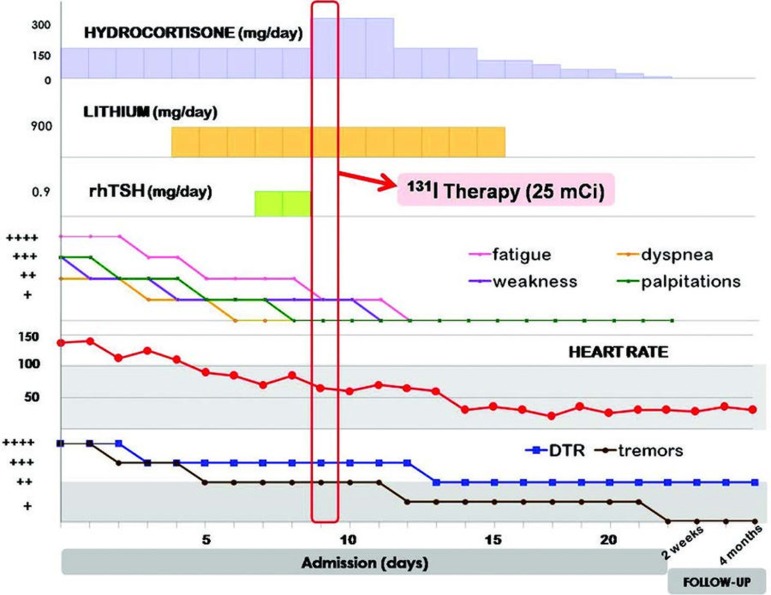
Course of Hydrocortisone, Lithium, rhTSH, and Subsequent Clinical Improvement DuringAdmission for Iodine-131Therapy and Follow-up. Shaded area for heart rate and deep tendon reflexes, tremors represents normal range.

Course after RAI (Figure 1): There was a subsequent improvement in the patient’s symptoms, deep tendon reflexes (DTR), heart rate, , and tremors. She had a transient further elevation of ALT two days after rhTSH and 1 day after RAI treatment, without accompanying jaundice. ALT levels improved thereafter. One week after RAI, free T4 levels were elevated (30.2 pmol/L) and free T3 levels were normal (5.2 pmol/L), and there was no recurrence or worsening of symptoms/signs of hyperthyroidism. She was eventually discharged and her condition was significantly improved. Four weeks after RAI, the level of TSH was still suppressed (0.005 uIU/mL, n = 0.27-4.2) and free T4 levels were already normal (16.85 pmol/L, n = 12-22). Shewas subsequently rendered hypothyroid. She is presently clinically euthyroid, on thyroid hormone replacement and is back at work as a grade school teacher. We obtained written informed consent for the management of her extraordinary case.

## 3. Discussion

The clinical presentation of our patient prior to amiodarone administration suggested Graves’ thyrotoxicosis, complicated by potential fatal tachyarrhythmias in the background of thionamide withdrawal secondary to agranulocytosis. She was given amiodarone for cardio version after resuscitation from cardiorespiratory arrest. Whether she developed amiodarone-induced thyrotoxicosis (AIT) thereafter, is difficult to establish. When she presented at our institution she had features that were indistinguishable between spontaneous hyperthyroidism and AIT, and type 1 AIT frequently occurs in patients with established thyroid dysfunction (13). Amiodarone is a potent class III anti-arrhythmic drug widely used for the treatment of supraventricular and ventricular arrhythmias. It is an iodine-rich benzo furanic derivative (approximately 37% iodine by weight) whose structural formula closely resembles that of T4. In peripheral tissues, amiodarone inhibits type I 5’-deiodinase (5’-D) activity, resulting ininhibition of the monodeiodination outer ring of T4 to generate T3. Amiodarone and its metabolite desethylamiodarone likewise exert cytotoxic effects on the thyroid.

Studies in rats showed amiodarone-associated ultra structural changes indicative of; thyroid cytotoxicity, marked distortion of thyroid architecture, apoptosis, necrosis, inclusion bodies, lipofuscinogenesis, macrophage infiltration, and markedly dilated endoplasmic reticulum, which were distinct from those induced by excess iodine alone (14). This effect probably contributes to the associated destructive thyroiditis with amiodarone. Moreover, excess iodide induces apoptosis through p53-independent oxidative stress, associated with production of reactive oxygen species and an increase in lipid peroxide levels. Iodide excess can also decrease sodium-iodide symporter (NIS) with subsequent decrease in the thyroid iodide trap. In normal rats exposed to high, chronic doses of iodide, NIS proteins and mRNA are markedly decreased. Altogether, these mechanisms may result in low or decreased radioactive iodine uptake. Low thyroidal RAIU is likewise associated with iodine excess, due to the dilution of the radioisotope by the increased stableiodide pool, especially in hyperthyroid patients (15).

After 10 days on amiodarone therapy, our patient demonstrated low RAIU (6% at 0400h and 7% at 2400h), presumably because of the combined destructive and cytotoxic effects of the drugs and the effects of the iodine-rich milieu on apoptosis, NIS expression and dilution of radioisotope. Although our patient had features suggestive of the thyroidal effects of amiodarone, low T3 with normal T4 and low RAIU levels, the precise diagnosis of AIT is very complex especially in this particular case. AIT can present as iodine-induced thyrotoxicosis (AIT type 1) or destructive thyroiditis (AIT type 2), but her features; underlying thyroid disease, painless thyroid, normal CFDS, low RAIU, mild clinical improvement on glucocorticoids, suggested a mixed/indeterminate type. The presence of thyroid antibodies and response to specific therapy may be helpful in distinguishing which pathogenesis is more predominant. While it is crucial to determine which type of AIT may have developed before initiating the appropriate therapy, we would like to point out two important events in her history, she had thionamide-induced agranulocytosis (thionamide is given for type 1 AIT), and she was already on steroids when she was referred to our center (glucocorticoids are preferred for type 2 AIT). These posed a further challenge with regards to how to render her euthyroid, as she could have developed fatal tachyarrhythmias again at anytime, while still thyrotoxic. Faced with such a predicament and pressed for time, we decided to manage her case as Graves’ thyrotoxicosis, complicated by amiodarone-induced low RAIU. Hence, we considered combining rhTSH (which reportedly increasesiodine-131 uptake) and lithium (which enhances retention of iodine-131) to obtain possible satisfactory RAIU and potentially facilitate RAI treatment of her thyrotoxicosis. The RAI therapy was directed at her Graves’ disease with the goal of rendering her hypothyroid, and the combination regimen of rhTSH plus lithium was intended to increase her low RAIU levels. We cannot categorically say at this point that our treatment protocol is for the treatment of AIT or Graves’ with AIT.

Our regimen of rhTSH in combination with lithium resulted in a more than five-fold increase of iodine-131 uptake in our patient with low RAIU secondary to amiodarone therapy. We are not certain which of the two drugs affected the RAI uptake of the thyroid the most. In thyroid follicular cells, the Na+/I-symporter (NIS) catalyzes iodide uptake across the basolateral membrane. rhTSH significantly increases NIS messenger RNA in FRTL-5 cells, reaching a maximum at 24 hours. However, NIS protein levels were significantly increased after 36 hours, reaching a maximum at 72 hours, therefore, optimal expression and/or activation of the NIS may require some additional time. Indeed, a 24-hour interval betweenrhTSH administration and radioiodide administration was significantly more effective in increasing RAIU than a two hour interval (8). However, lithium inhibits thyroidal hormone release in hyperthyroid patients more than in normal patients, enhancing retention of RAI and thus it has been used as an adjuvant in RAI with Graves’ disease with low RAIU (2, 5).

This is the first case in the Philippines to demonstrate the efficacy of rhTSH in improving RAIU in an iodine-loaded patient with thyrotoxicosis. Lawrence et al. (2001) showed that a single dose of 0.9mg rhTSH moderately increased thyroid RAIU (88%) in normal subjects with depressed iodine-131 uptake, after iodide administration in normal subjects (16). The utility of rhTSH stimulation prior to RAI therapy of elderly type 1 AIT patients have likewise been reported, albeit with caution because of increased thyroid hormones and atrial arrhythmias after RAI (12). In contrast to the clinical profile of previously reported cases (elderly with multi nodular goiter and elevated free T4 and free T3), our patient was relatively young and had a diffusely enlarged goiter, with low baseline thyroid hormone levels.

Our experience demonstrates the efficacy and safety of rhTSH plus lithium as an adjuvant to RAI therapy in patients with suppressed TSH and low RAIU, particularly in patients who are young and without a previous history of cardiac disease.
